# Infarct of the Middle Cerebellar Peduncle Mimicking Bell’s Palsy: A Case Report

**DOI:** 10.7759/cureus.67533

**Published:** 2024-08-22

**Authors:** Uthayanila Pandian, Arun K, Subramaniyan Kumarasamy, J S Kumar, Aamina Hussain

**Affiliations:** 1 General Medicine, SRM Medical College Hospital and Research Centre, SRM Institute of Science and Technology, Chengalpattu, IND; 2 General Medicine, Karpaga Vinayaga Institute of Medical Sciences, Chennai, IND; 3 Community Medicine, SRM Medical College Hospital and Research Centre, SRM Institute of Science and Technology, Chengalpattu, IND

**Keywords:** anterior inferior cerebellar artery syndrome, bell's palsy, lower motor neuron palsy, middle cerebellar peduncle infarct, anterior inferior cerebellar artery

## Abstract

The anterior inferior cerebellar artery (AICA) supplies the middle cerebellar peduncle, lower pons, upper medulla, and anterior inferior cerebellum. Ischemia in the AICA can cause the lateral inferior pontine syndrome. AICA syndrome is characterized by facial sensory loss and weakness, Horner syndrome, prolonged vertigo, audio-vestibular loss, and cerebellar signs. Many studies on AICA territory infarcts have demonstrated the rarity of complete AICA syndrome. In all cases of AICA territory infarcts, involvement of the middle cerebellar peduncle was observed, with the seventh cranial nerve (facial nerve) being the most frequently involved cranial nerve, vertigo was the most common presenting symptom, and atherosclerosis was the most common etiology. This case report aims to investigate the occurrence of middle cerebellar peduncle infarcts that mimic Bell’s palsy, highlighting the importance of accurate diagnosis and appropriate management in such cases. Recognizing the unique characteristics and clinical presentation of middle cerebellar peduncle (MCP) infarcts is essential for distinguishing them from more common conditions like Bell’s palsy, thereby ensuring timely and effective treatment.

## Introduction

The cerebellum, located over the posterior aspect of the pons and medulla, plays a crucial role in motor control and coordination. The ratio of afferent to efferent pathways in the cerebellum is approximately 40:1, with the superior cerebellar peduncle serving as the primary efferent pathway and the middle cerebellar peduncle (MCP) being the main afferent pathway, composed of white matter fibers originating from contralateral pontine nuclei [1]. The middle cerebellar peduncle infarct (MCP) is a watershed area between the supply of the anterior inferior cerebellar artery (AICA) and the superior cerebellar artery, making it susceptible to hypoperfusion. Infarction in this region indicates hypoperfusion, often involving the middle cerebellar peduncle in almost all cases [2].

Isolated infarcts of the middle cerebellar peduncle are uncommon, with the most common lesions being related to myelin abnormalities and pontine ischemia or hemorrhage causing Wallerian degeneration [3]. An isolated AICA infarction due to thromboembolism or atherosclerosis shows restricted diffusion in the middle cerebellar peduncle. An AICA infarct can involve the middle cerebellar peduncle alone or in conjunction with the lower lateral pons and anterior inferior cerebellum. Clinical syndromes associated with AICA may originate from cardioembolic or atherosclerotic causes, typically secondary to hypertension or other risk factors affecting its vascular territory [4].

Involvement of the motor fibers of the facial nerve in an AICA infarct can lead to various symptoms such as loss of forehead creases, inability to raise the eyebrow, deviation of the angle of the mouth, loss of the nasolabial fold, and a positive Bell's phenomenon, as seen in our case. In a study of 82 patients with AICA territory infarction, 98% presented with vertigo, 5% had isolated vestibular infarction without cochlear involvement, and only 1% had no other central symptoms or signs, such as facial nerve palsy or sensory loss. Subsequent studies have also demonstrated the frequent involvement of the facial nerve and prolonged vertigo in patients with AICA territory infarct [4,5].

Although complete AICA syndrome is rare, a recent review indicated that 10% to 31% of patients with an infarct in the AICA distribution can experience preceding audio-vestibular disturbances such as vertigo, nausea, ataxia, and hypoacusis before the onset of other neurological signs. These patients can also initially present with negative brain imaging [6]. The AICA supplies the middle cerebellar peduncle, lateral tegmentum of the lower pons and upper medulla, flocculus, and anterior inferior cerebellum. Ischemia in the AICA territory causes lateral inferior pontine syndrome [7].

Patients with cerebellar dysfunction may present with tremor, incoordination, nausea, nystagmus, and vertigo, with ataxia being the cardinal sign of cerebellar disease. Involvement of the complete AICA can result in lateral pontine syndrome, characterized by sudden onset vertigo, nausea, vomiting, nystagmus, ipsilateral ataxia, lower motor neuron facial weakness with loss of taste, sensation, and salivation on the ipsilateral side, hearing loss and tinnitus on the ipsilateral side, loss of pain and temperature sensation on the ipsilateral side of the face, and Horner syndrome [7,8]. Bell's palsy, the most common cause of seventh cranial nerve paralysis, is a self-limiting unilateral facial paralysis with acute onset, usually occurring between the ages of 15 and 45 years [9]. In contrast, strokes involving the upper motor neuron type facial nerve palsy typically affect one quadrant of the face rather than half of the face, as seen in Bell's palsy. However, in cases of brainstem infarction primarily involving the pons, patients can present with unilateral facial nerve paralysis of the lower motor neuron type [10].

This case report aims to study the occurrence of middle cerebellar peduncle infarctions that mimic Bell's palsy, highlighting the need for precise diagnosis and appropriate management in such situations. Assessing the unique features and clinical presentation of the middle cerebellar peduncle (MCP) infarctions is critical for separating them from more frequent illnesses, such as Bell's palsy, allowing for earlier and more efficient treatment.

## Case presentation

A 43-year-old Indian male patient with a known history of systemic hypertension for the previous four years arrived at the emergency room complaining of deviation of the angle of his mouth to the left side and slurring of speech during the previous 24 hours. There was no documented history of fever, recent infection, skin lesions, or cold exposure. Upon additional questioning, the patient revealed episodes of giddiness, nausea, and vomiting beginning five days before the onset of facial weakness. Importantly, there was no reported history of focal limb weakness or sensory disturbances, and the patient denied any head trauma or illicit drug use.

**Figure 1:**
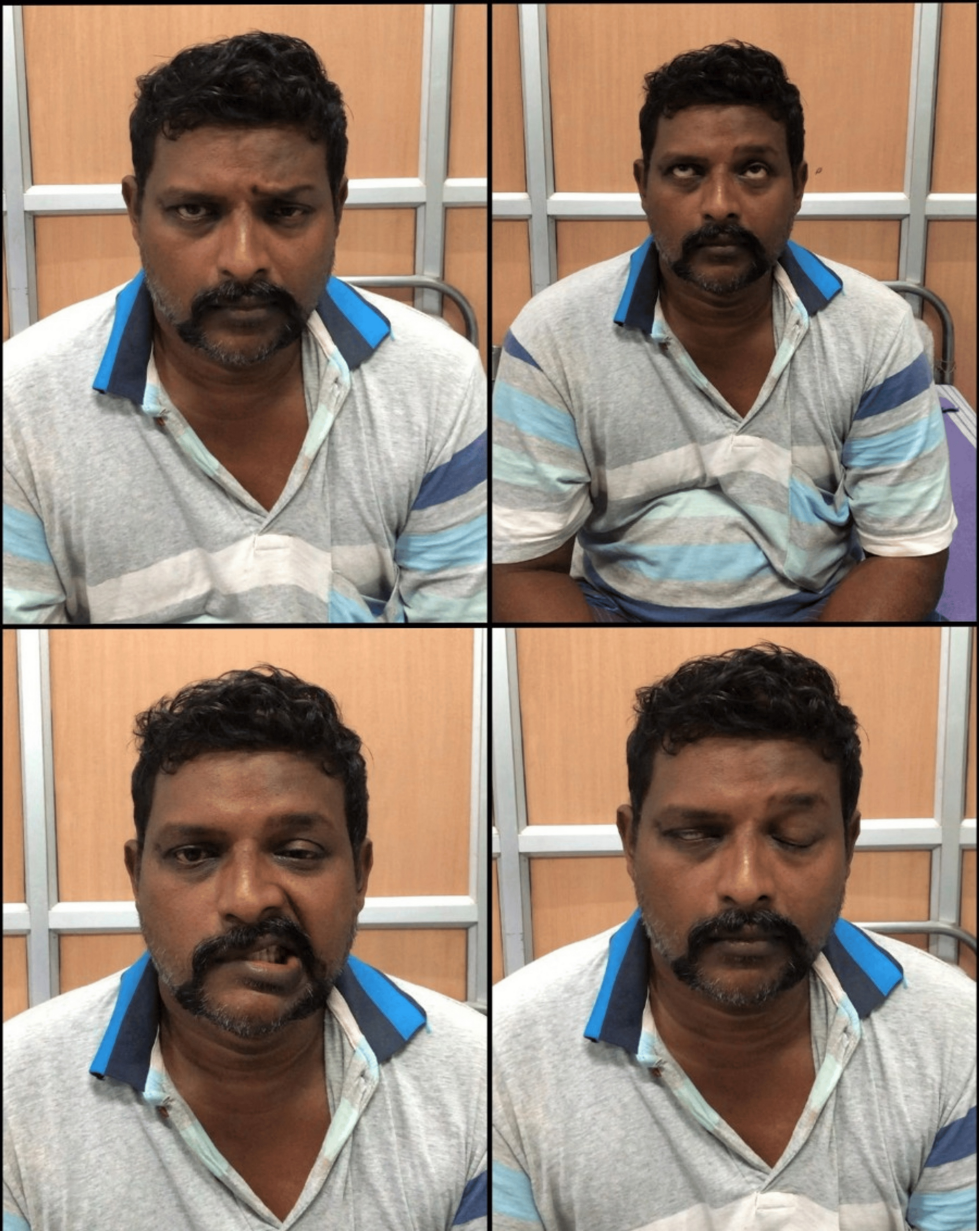
Positive Bell's phenomenon left-top: shows absence of forehead crease in right side; right-top: inability to lift right eyebrow; left -bottom: angle of mouth deviated to left on smiling; right-bottom: positive Bell’s phenomenon in right eye

The patient had undergone an initial CT scan five days earlier during the onset of giddiness, which returned negative results. At the time of presentation, the patient remained conscious and alert, without fever, with blood pressure measuring 130/70 mmHg and a heart rate of 78 beats per minute. On examination, the findings are presented in Table 1 and Figure 1.

**Table 1:** Examination findings

S.NO	Examination	Findings
1.	Neurological examination	No objective motor or sensory abnormalities in either the upper or lower limbs, nuchal rigidity was notably absent.
2.	Cranial nerve examination	Right facial paralysis of the lower motor neuron type, characterized by the following:
		a) Loss of the forehead crease on the right side
		b) Inability to raise the eyebrow on the right side
		c) Deviation of the mouth angle to the left side
		d) A positive Bell's phenomenon was observed on the right side as seen in Figure 1
		Other cranial nerve examinations yielded normal results
3.	Cerebellum examination	Cerebellar ataxia swaying to the right during tandem walking
		No presence of nystagmus
		No dysdiadochokinesia

**Figure 2:**
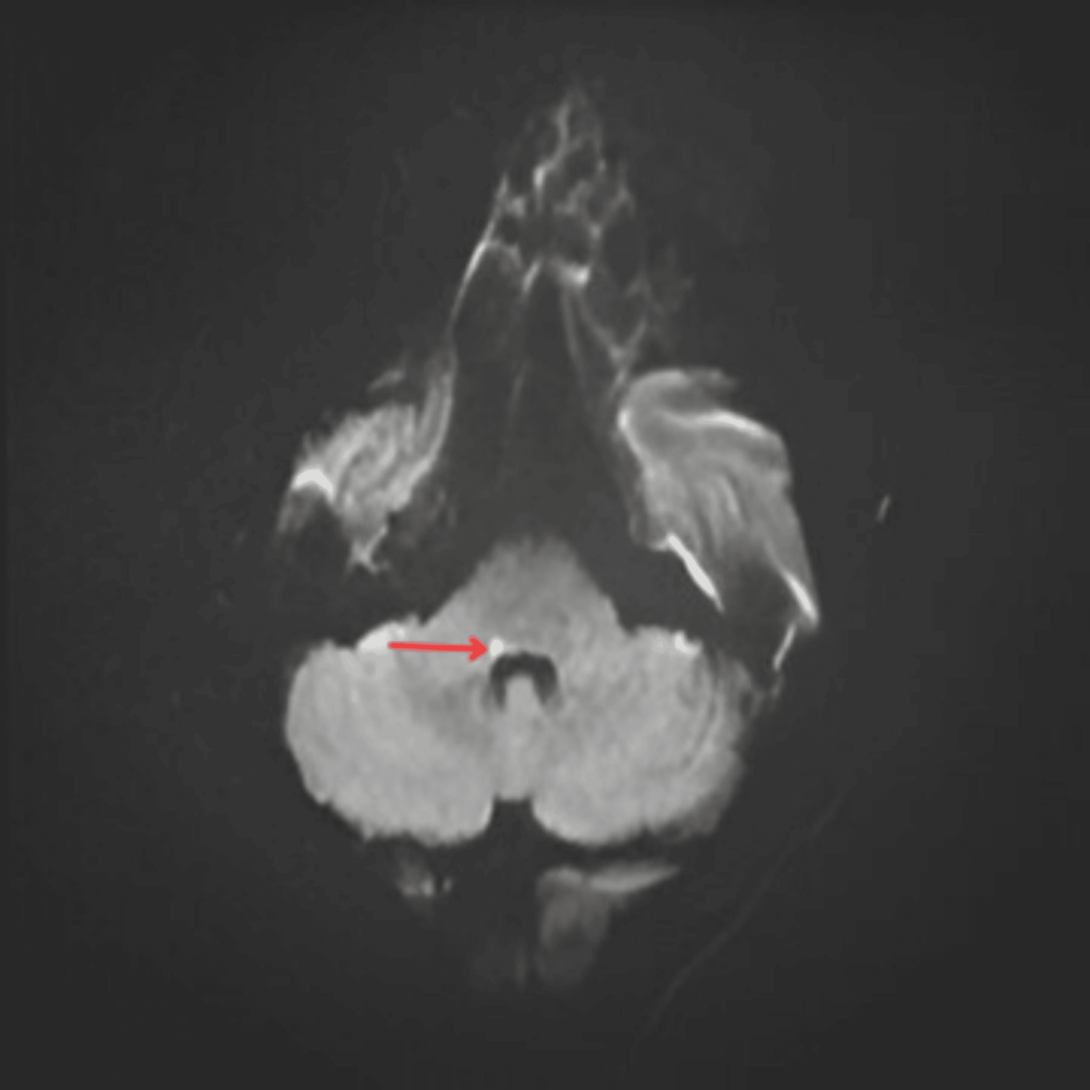
Magnetic resonance image (MRI) brain with hyperintense foci at junction of middle cerebellar peduncle (MCP) with lentiform nucleus (LPT) (floor of fourth ventricle) in diffusion-weighted imaging (DWI) Arrow in the figure indicates area of infarct

**Figure 3:**
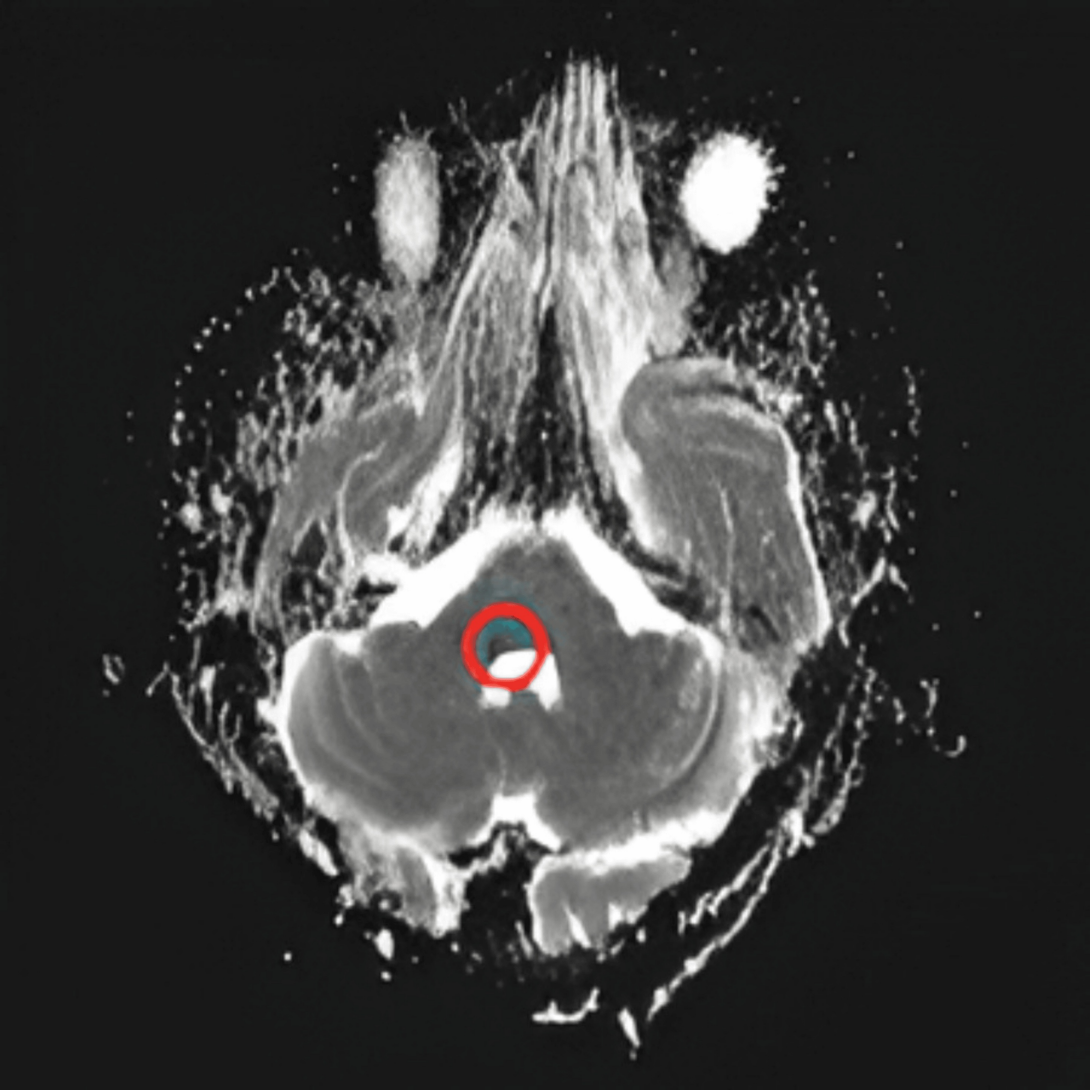
Magnetic resonance imaging (MRI) brain with hypointense foci noted in same area in apparent diffusion coefficient (ADC), confirming true diffusion restriction suggestive of infarct ⭕ in the figure indicates the area of infarct

**Figure 4:**
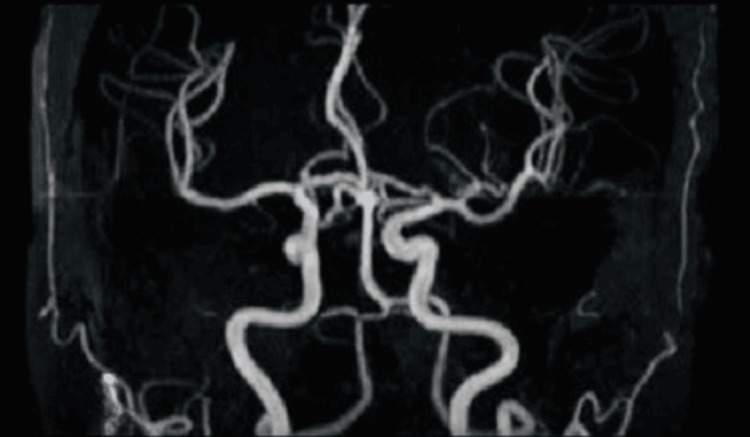
Magnetic resonance (MR) angiography showing normal findings

The patient had received initial treatment five days earlier, consisting of normal saline, ondansetron, promethazine, and betahistine, which successfully alleviated symptoms of giddiness, and the initial investigations returned within normal ranges (Table 2). Given the inability to definitively rule out acute ischemic stroke through computed tomography scans (CT) alone, a plain magnetic resonance imaging (MRI) of the brain was conducted, initially showing no abnormalities. However, due to the patient's progression to facial nerve paralysis after six days, along with other neurological signs such as ataxia, a re-evaluation of the imaging after seven days revealed findings mentioned in Table 3 and Figures 2, 3, 4.

**Table 2:** Investigation findings

Investigation	Result	Reference value
White blood cell count	10570	4000-11000
Hemoglobin	14g/dl	13 g/dl-17g/dl
Platelet count	3,24,000	1,50,000 - 4,00,000
Neutrophil	78.6%	40%-80%
Lymphocyte	15.3%	20%-40%
Monocyte	3%	2%-10%
Mean corpuscular volume (MCV)	74fl	83fl-101fl
Mean corpuscular hemoglobin (MCH)	23pg	27pg-32pg
Mean corpuscular hemoglobin concentration (MCHC)	31g/dl	31.5g/dl-34.5g/dl
Sodium	133 mmol/L	136 mmol/L-140mmol/L
Chloride	100mmol/L	98 mmol/L -107 mmol/L
Potassium	4.1 mmol/L	3.5 mmol/L -5.1 mmol/L
Bicarbonate	25mmol/L	21 mmol/L -31 mmol/L
Total cholesterol	167 mg/dL	<170 mg/dL
Low-density lipoprotein (LDL)	122 mg/dL	<100 mg/dL
High-density lipoprotein (HDL)	29 mg/dL	>45 mg/dL
A hemoglobin A1C (HbA1c)	11.0%	<6%
Serum urea	18mg/dl	17 mg/dL -43 mg/dL
Serum creatine	0.9mg/dl	0.5 mg/dL - 1.1 mg/dL

**Table 3:** Investigation findings

Investigation	Results
Electrocardiogram (ECG)	Normal
Magnetic resonance imaging (MRI) brain	A small non-hemorrhagic lacunar infarct at the junction of the MCP and lateral pontine tegmentum. The lesion exhibited hyperintense foci in diffusion-weighted imaging DWI (Figure 2) and hypointense foci in apparent diffusion coefficient (ADC) (Figure 3), indicative of true diffusion restriction and suggestive of infarction.
Magnetic resonance (MR) angiography of the head and neck	No significant findings (Figure 4)
Transesophageal echocardiogram	No significant findings

After a five-day hospital stay, the patient was discharged on dual antiplatelet and statin therapy for a 21-day course. Blood pressure was maintained under control with a calcium channel blocker. The patient was advised to restrict salt intake, review with a physical therapist and speech therapist, and instructed to return for a review after three weeks for a potential change from dual antiplatelet therapy to a single agent.

## Discussion

Many studies suggest that around 7% of all ischemic strokes are pontine infarcts, while 15% of all posterior circulation infarcts are caused by isolated pontine strokes [11]. The majority are lacunar infarcts affecting small vessels in the posterior circulation, such as the basilar artery perforators [12], with hypertension being a significant risk factor [11,12]. Isolated infra-nuclear facial paralysis is often caused by a lacunar infarct involving the lower pons.

Studies have shown that rare pontine strokes affecting the facial nerve tract or ipsilateral nucleus before the facial nerve leaves the pons indicate a central lesion that mimics a peripheral lesion. Bell's palsy usually develops over several hours to days and is not typically accompanied by nausea, vomiting, dizziness, or diplopia. In contrast, these insults happen suddenly.

In our case, the patient presented with right facial paralysis of the lower motor neuron type, characterized by the loss of the forehead crease on the right side, inability to raise the eyebrow on the right side, and deviation of the mouth angle to the left side. A positive Bell's phenomenon was observed on the right side, and the patient has a well-recognized risk factor for stroke: hypertension. Facial weakness can be caused by a variety of conditions, including peripheral causes like Bell's palsy and central reasons such as strokes or tumors. In this case, the presence of lower motor neuron-type facial paralysis and cerebellar ataxia suggested a central cause, especially given the patient's history of hypertension. According to the literature, pontine strokes can cause isolated facial nerve palsy, which is frequently misdiagnosed as Bell's palsy. This case emphasizes the importance of a thorough clinical evaluation and the possibility of cerebrovascular events in patients with severe facial paralysis, particularly when accompanied by atypical systemic symptoms.

A case report describes a 39-year-old female who presented with vertigo, nausea, and vomiting as the initial complaint. The key similarities between both patients are the stepwise presentation of symptoms and the surprising presentation of complete unilateral facial paralysis mimicking Bell's palsy [13]. Adding to this, there was difficulty in identifying the infarction on CT in both cases. Studies have found that MRI diffusion-weighted imaging (DWI) is more effective than CT scans in identifying acute ischemic stroke; however, even DWI might result in false-negative results within the initial twenty-four hours following the presentation. These false-negative results are more common in strokes affecting the brainstem and posterior circulation. Oppenheim et al. [14] observed that 5.8% of the 139 stroke patients in their study had negative MRI results within the first 24 hours of presentation. Such lesions that are initially negative on MRI may be detected with a repeat MRI conducted 24 hours after the onset [14].

In our case, the initial CT scan revealed no abnormalities, which is not unusual in early ischemic strokes. Following MRI, hyperintense foci in diffusion-weighted imaging (DWI) and hypointense foci in apparent diffusion coefficient (ADC) indicated the ischemic insult in the right middle cerebellar peduncle and adjacent floor of the fourth ventricle (formed by lateral pontine tegmentum), resulting in complete hemi-facial paralysis imitating Bell's palsy. These imaging characteristics are critical for distinguishing between ischemic and hemorrhagic strokes and locating the infarct. The diagnosis of the lesion at the MCP is especially important since it might cause both cerebellar symptoms and facial nerve involvement due to the physical proximity of the affected tissues. This case demonstrates the importance of using modern imaging modalities in unclear clinical presentations to guarantee correct diagnosis and timely action.

The patient was treated with dual antiplatelet therapy and statins, as recommended by current stroke secondary prevention guidelines. This approach is essential for people with vascular risk factors because it minimizes the likelihood of repeated cerebrovascular incidents. The patient's discharge plan included lifestyle adjustments such as dietary changes and follow-up with rehabilitation programs, both of which are essential components of complete stroke care. The prognosis for lacunar infarctions is generally favorable, especially when modifiable risk factors such as diabetes mellitus, smoking, high blood pressure, and hyperlipidemia are well managed. Patients with a history of myocardial infarction have a lower risk of stroke when taking statins. Early detection and treatment of underlying vascular problems may considerably enhance results and lower the risk of future strokes.

When clinical or hematological signs indicate thrombophilia, a thrombophilia screen is required. Blood cultures should be performed if subacute bacterial endocarditis or a brain abscess are suspected. Even if the initial MRI or CT scan of the head contradicts the physician's clinical suspicion, a high degree of clinical suspicion is still necessary. This case highlights the challenges of identifying facial weakness as well as the vital significance of considering a wide range of causes, including central etiologies, particularly in patients with vascular risk factors. Clinicians should keep a high suspicion for cerebrovascular events in patients who report with unusual facial paralysis symptoms, since early intervention can have a major impact on patient outcome. This case serves as a sobering reminder of the value of anatomical understanding as well as the challenges associated with diagnosing a pontine stroke [15].

## Conclusions

It is pivotal to rapidly identify stroke to avoid morbidity and mortality in patients with acute cerebrovascular accidents. This case emphasizes the importance of a thorough central nervous system examination in any patient with facial palsy. Acute cerebrovascular accidents causing stroke should be considered as a differential diagnosis for Bell’s palsy when patients exhibit central neurological signs and symptoms. 

This case highlights the importance of modern imaging tools, such as MRI, in accurately detecting cerebrovascular events with unique clinical characteristics. By recognizing the possibility of central causes of facial weakness, physicians can avoid misdiagnosis and ensure timely and proper treatment, ultimately improving patient outcomes.

Additionally, this case emphasizes the importance of thorough evaluation in patients with vertigo without typical inner ear disorders. Overall, this report serves as a valuable reminder of the difficulties inherent in neurological examinations and the significance of remaining vigilant in clinical practice when dealing with misleading presentations. It asserts the need for a thorough evaluation of patients with facial paralysis to promote early intervention and limit the risk of future neurological problems.
